# Years of life lost to prison: racial and gender gradients in the United States of America

**DOI:** 10.1186/1477-7517-5-4

**Published:** 2008-01-25

**Authors:** Robert S Hogg, Eric F Druyts, Scott Burris, Ernest Drucker, Steffanie A Strathdee

**Affiliations:** 1 Faculty of Health Sciences, Simon Fraser University, Burnaby, British Columbia, Canada; 2 British Columbia Centre for Excellence in HIV/AIDS, St. Paul's Hospital, Vancouver, British Columbia, Canada; 3 Division of International Health and Cross Cultural Medicine, San Diego School of Medicine, University of California, California, USA; 4 Montefiore Medical Center, New York, New York, USA; 5 Beasley School of Law, Temple University, Philadelphia Pennsylvania, USA

## Abstract

**Background:**

The United States has the highest rate of imprisonment of any country in the world. African Americans and Hispanics comprise a disproportionately large share of the prison population. We applied a "prison life expectancy" to specify differences in exposure to imprisonment by gender and race at the population level.

**Methods:**

The impact of imprisonment on life expectancy in the United States was measured for each year from 2000 to 2004, and then averaged. Using the Sullivan method, prison and prison-free life expectancies were estimated by dividing the years lived in each age range of the life table into these two states using prevalence of imprisonment by gender and race.

**Results:**

African American males can expect to spend on average 3.09 years in prison or jail over their lifetime and Hispanic and Caucasian males can spend on average 1.06 and 0.50 years, respectively. African American females, on the other hand, can expect to spend on average 0.23 years in these institutions and Hispanic and Caucasian females can expect to spend on average 0.09 and 0.05 years, respectively. Overall, African American males, the highest risk group, can expect to spend on average 61.80 times longer in prison or jail as compared to Caucasian women, the lowest risk group.

**Conclusion:**

There are clear gender and racial gradients in life expectancy spent in prison in the United States. Future research needs to examine how current imprisonment practice in the United States may influence population health and health disparities.

## Introduction

The United States has the highest rate of incarceration in the developed world. Nearing the end of the 1990s, over two million people were behind bars and another four and half million people were on probation or parole. The number of people imprisoned almost doubled in the 1990s, increasing from one in every 218 residents in 1990 to one in every 145 in 2001 [1]. Imprisonment has not been evenly distributed throughout the population. Prison populations are comprised of disproportionate numbers of African Americans and Hispanics. The lifetime probability of being imprisoned in 2001 was six times higher for males than females – 11.3% versus 1.8%. Among males, African Americans have a one in three chance of being imprisoned during their lifetime, whereas Hispanics and Caucasians have a one in six and a one in 17 chance, respectively [1,2].

Life expectancy is an essential indicator of population health. It may be refined by techniques that assess the quality of expected life years, such as the disability-free life expectancy. The high rate of imprisonment in the United States may translate to a significant proportion of time being spent in prison, especially for certain sub-groups of the population. If imprisonment influences life expectancy, time spent in prison becomes a matter of public health importance. The goal of the present study was to determine the differences in the number of years of life lost to imprisonment in the United States population by gender and race.

## Methods

The number of years of life lost to imprisonment in the United States was measured for each year from 2000 to 2004, and then averaged for this time period. Population data were derived from the US Census Bureau, National Population Estimates [3], and the proportion of the total population imprisoned at mid-year in jails and prisons were estimated from data obtained from the US Department of Justice for the years 2000 to 2004 [4-8]. Life tables were obtained from US Census Bureau, National Population Projections [9].

To provide context, rates of imprisonment and person-years of life lost to imprisonment were first calculated. Rates were calculated for each year, 2000 through 2004, for the age group 18 to 44 years. Rates of imprisonment in this age group were the average of the number of persons in prison per 100,000 population. Person-years of life lost to imprisonment were calculated by multiplying the number of persons imprisoned in a specific age group by years left to 45 years. Person years were then totaled for each gender and racial group and expressed as person years lost per 100,000 population.

The Sullivan method [10] was used to estimate the impact of imprisonment on life expectancy in the United States. Sullivan's method involves using the prevalence of health states at each age in the current population (at a given point of time) to divide the hypothetical years of life lived by a period life table cohort at different ages into years with and without disability. In our example, prison (disability) and prison-free life expectancies were estimated by dividing the years lived in each age range of the life table into these two states using prevalence of imprisonment by gender and race. These figures were then used to compute the life table value of the total remaining years of life and the corresponding life expectancy in each state for each age group. Total life expectancy at birth or any other age group by race and gender was the sum of life expectancy in the prison and prison-free states.

## Results

There was on average 1.75 million persons between the ages of 18 and 44 in prison in the United States between 2000 and 2004. The vast majority of prisoners over this time period were male (92.5%). African American males comprised the largest percentage of the male prison population (45.2%). Caucasian and Hispanic males constituted 34.3% and 18.4% of the total male prison population, respectively. Among the female imprisoned population, Caucasian and African American females comprised the largest percentages (40.9% and 41.7%, respectively) followed by Hispanic females (14.5%) (Table 1).

**Table 1:** Number of persons in prison in the United States, 2000–2004, by race and gender, age 18–44

Year	Total*	Caucasian n (%)	African American n (%)	Hispanic n (%)
Female				

2000	141,100	56,300 (39.9)	64,200 (45.5)	17,600 (12.5)
2001	146,500	60,100 (41.0)	64,200 (43.8)	18,100 (12.4)
2002	146,600	59,700 (40.7)	58,500 (39.9)	22,800 (15.6)
2003	152,500	65,100 (42.7)	58,100 (38.1)	24,800 (16.3)
2004	157,900	69,700 (44.1)	58,400 (37.0)	25,300 (16.0)
**2000–2004**	**148,920**	**62,180 (41.7)**	**60,680 (40.9)**	**21,720 (14.5)**

Male				

2000	1,559,900	556,300 (35.7)	716,100 (45.9)	260,900 (16.7)
2001	1,592,200	578,000 (36.3)	729,600 (45.8)	257,500 (16.2)
2002	1,614,200	526,600 (32.6)	731,700 (45.3)	308,200 (19.1)
2003	1,612,000	534,800 (33.2)	719,700 (44.7)	322,000 (20.0)
2004	1,638,100	555,400 (33.9)	722,100 (44.1)	324,000 (19.8)
**2000–2004**	**1,603,280**	**550,220 (34.3)**	**723,840 (45.2)**	**294,520 (18.4)**

SOURCE: AJ Beck, JC Karberg. Prison and Jail Inmates at Midyear 2000, 2001, 2002, 2003, 2004.

*Includes Indians, Alaska Natives, Asians, Native Hawaiians, and other Pacific Islanders; totals for Caucasian, African American, and Hispanic will not equal to 100 percent.

Table 2 shows rates of imprisonment and person-years of life lost to prison in the United States, 2000–2004, by race and gender, for those aged 18–44. Rates of imprisonment were consistently highest among African Americans for either gender in all years. The average rate of imprisonment for ages 18 to 44 years ranged from 9,800 per 100,000 population in African American males to 165 per 100,000 population in Caucasian females over the time period. African American males also consistently lost the most person years of life between 18–44 years due to imprisonment over this time period. The average was nearly 140,000 years of life lost per 100,000 population compared to 1,229 years of life lost per 100,000 population for Caucasian females. In both males and females, there was a consistently clear gradient with rates for Hispanics being intermediate between those of African Americans and Caucasians for all years.

**Table 2:** Rates of imprisonment and person-years of life lost to prison in the United States, 2000–2004, by race and gender, age 18–44 (per 100,000  population)

Year	Caucasian		African American		Hispanic	
			
	Rate of imprisonment	Person-years of life lost to Prison	Rate of imprisonment	Person-years of life lost to Prison	Rate of imprisonment	Person-years of life lost to Prison
Female						

2000	152	1,983	811	10,341	225	3,186
2001	164	2,147	808	10,343	224	3,172
2002	163	2,073	732	8,898	275	3,843
2003	181	2,239	726	8,814	281	3,656
2004	165	1,738	726	8,823	289	3,917
**2000–2004**	**165**	**2,036**	**761**	**9,444**	**260**	**3,555**

Male						

2000	1,481	19,812	9,885	141,108	2,952	44,211
2001	1,549	20,865	9,975	141,602	2,795	42,259
2002	1,420	18,836	9,887	141,929	3,212	48,094
2003	1,454	18,961	9,656	136,808	3,238	47,659
2004	1,521	19,852	9,605	136,089	3,163	46,607
**2000–2004**	**1,485**	**19,665**	**9,800**	**139,507**	**3,078**	**45,766**

Table 3 shows years of life lost to imprisonment in the United States, 2000–2004, by race and gender. Males spent a greater proportion of their life in prison or jail than females. Considering either gender, African Americans spent much more of their life imprisoned than Hispanics and Caucasians. Based on these data, African American males can expect to spend on average 3.09 years in prison or jail over their lifetime and Hispanic and Caucasian males can spend on average 1.06 and 0.50 years, respectively. African American females, on the other hand, can expect to spend on average 0.23 years in these institutions and Hispanic and Caucasian females can expect to spend on average 0.09 and 0.05 years, respectively. Overall, African American males, the highest risk group, can expect to spend on average 61.80 times longer in prison or jail as compared to Caucasian women, the lowest risk group.

**Table 3:** Years of life lost to imprisonment in the United States, 2000–2004, by race and gender

Year	Caucasian			African American			Hispanic		
			
	Total	Non-prison component	Prison component	Total	Non-prison component	Prison component	Total	Non-prison component	Prison Component
Female									

2000	79.78	79.73	0.05	74.79	74.55	0.24	82.13	82.06	0.08
2001	79.89	79.84	0.05	74.98	74.74	0.24	82.13	82.06	0.07
2002	80.00	79.94	0.05	75.16	74.93	0.23	82.25	82.16	0.09
2003	80.10	80.05	0.06	75.34	75.11	0.23	82.31	82.21	0.10
2004	80.21	80.15	0.05	75.52	75.29	0.23	82.35	82.25	0.10
**2000–2004**	**80.00**	**79.94**	**0.05**	**75.16**	**74.92**	**0.23**	**82.23**	**82.15**	**0.09**

Male									

2000	74.92	74.42	0.50	68.58	65.50	3.08	76.66	75.62	1.05
2001	75.02	74.50	0.52	68.76	65.73	3.04	76.66	75.72	0.94
2002	75.12	74.65	0.47	68.95	65.86	3.09	76.85	75.75	1.09
2003	75.23	74.73	0.50	69.14	66.03	3.11	76.94	75.82	1.12
2004	75.33	74.81	0.52	69.33	66.22	3.11	77.03	75.94	1.09
**2000–2004**	**75.13**	**74.62**	**0.50**	**69.00**	**65.91**	**3.09**	**76.83**	**75.77**	**1.06**

Note: Standard errors for estimates range from <0.001 to 0.003.

## Discussion

Our study contributes to evidence that the burden of imprisonment is not evenly distributed across gender and race in the United States population. Males spend a greater proportion of their life in prison or jail than females. Considering either gender, African Americans spend much more of their life in prison or jail than Hispanics or Caucasians. The burden, if not the disparity, would be even larger if we considered people confined under the jurisdiction of local authorities and people that were on parole or probation, since the latter group is currently twice the size of those imprisoned [11]. Furthermore, the percentage of time spent in prison is conservative in our study because the estimate of life expectancy is from birth, even though most people who are imprisoned are between 18 and 44 years of age.

Although data are limited, there is a growing concern that imprisonment can have serious negative health consequences [12,13]. Prison populations exhibit an elevated prevalence of communicable disease [14]. High levels of violence, including sexual violence, have been reported among imprisoned populations [15,16]. Consensual sex without condoms as well as drug injection and tattooing without sterile equipment are reported to occur at dangerous levels and to result in transmission of diseases, such as HIV [12,17-21]. Additionally, imprisonment may have life altering health consequences mediated by factors such as a decline in socio-economic status [22].

Our findings, and the growing literature on the negative health consequences of imprisonment, suggest that the extensive reliance on incarceration to control behavior in the United States has social costs that have not been fully recognized [22]. These social costs are not evenly distributed and may be contributing to population health disparities [23]. These health consequences may be reduced to some degree by making prisons more salubrious – by introducing better health, addiction treatment and mental health care, distributing condoms and needles, preventing violence and coordinating services as inmates move back into the community [12]. The most direct way to reduce these consequences would be to reduce the number of people who go to prison. One way to accomplish this would be by reducing the number of behaviors subject to imprisonment. Because laws criminalizing drug possession are the major driver of the imprisonment of the non-violent offenders in the United States, changes in these laws could be expected to have a significant impact. Other steps would include investing more in drug treatment, mental health care and other services that can forestall offending, reduce recidivism or serve as alternatives to imprisonment.

The use of mid-year sample data from the Bureau of Justice Statistics to measure the number of persons imprisoned may represent a limitation in our analyses. These data are based on sample estimates and do not reflect a complete census of prisoners. We must also recognize that these data exclude persons confined in locally administered facilities who are under the jurisdiction of local authorities and those who are on parole or probation [4]. Additionally, the Sullivan method does not reflect transitions in and out of prison. However, regardless of prison term or transitions in and out of prison, the potential health consequences identified above are still of concern.

More research on the health effects of imprisonment and new interventions to reduce them should be an urgent priority within both corrections and public health. Even in the absence of additional data, our study suggests that health consequences should be given greater weight in discussions of law enforcement strategies generally and drug policy in particular.

## Competing interests

The author(s) declare that they have no competing interests.

## Authors' contributions

RSH, SB, ED, and SAS initiated the study. RSH and EFD collected and analyzed the data. All authors contributed to the writing of the manuscript. All authors reviewed the final manuscript for important intellectual content.
